# Update on Evidence and Directions in Temporomandibular Joint Injection Techniques: A Rapid Review of Primary Research

**DOI:** 10.3390/jcm13144022

**Published:** 2024-07-10

**Authors:** Karolina Lubecka, Kamila Chęcińska, Filip Bliźniak, Maciej Chęciński, Natalia Turosz, Iwona Rąpalska, Adam Michcik, Dariusz Chlubek, Maciej Sikora

**Affiliations:** 1 Department of Oral Surgery, Preventive Medicine Center, Komorowskiego 12, 30-106 Kraków, Poland; lubeckarolina@gmail.com (K.L.); fblizniak@gmail.com (F.B.); maciej@checinscy.pl (M.C.); iwona.rzasa@uj.edu.pl (I.R.); 2 Department of Glass Technology and Amorphous Coatings, Faculty of Materials Science and Ceramics, AGH University of Science and Technology, Mickiewicza 30, 30-059 Kraków, Poland; checinska@agh.edu.pl; 3 Department of Maxillofacial Surgery, Hospital of the Ministry of Interior, Wojska Polskiego 51, 25-375 Kielce, Poland; natalia.turosz@gmail.com; 4 Chair of Oral Surgery, Medical College, Jagiellonian University, Montelupich 4, 31-155 Kraków, Poland; 5 Department of Maxillofacial Surgery, Medical University of Gdansk, Mariana Smoluchowskiego 17, 80-214 Gdańsk, Poland; adammichcik@gumed.edu.pl; 6 Department of Biochemistry and Medical Chemistry, Pomeranian Medical University, Powstańców Wielkopolskich 72, 70-111 Szczecin, Poland

**Keywords:** arthrocentesis, intra-articular injections, temporomandibular disorders, temporomandibular joint

## Abstract

This rapid review summarizes the latest primary research in temporomandibular joint (TMJ) injection treatment. The final literature searches were conducted on 4 January 2024. Selection was performed systematically following predefined eligibility criteria. Randomized control trials concerning the treatment of TMJ disorders with intra-articular injections were included. Studies on more invasive interventions were excluded. Quality of life, joint pain and range of mandibular mobility were assessed. Ultimately, 12 studies covering a total of 603 patients qualified. They concerned: (1) arthrocentesis (AC) and the administration of, (2) injectable platelet-rich fibrin (I-PRF), (3) platelet-rich plasma (PRP), (4) hyaluronic acid (HA), (5) non-steroidal anti-inflammatory drugs (NSAIDs), and (6) hypertonic dextrose (HD) with a local anesthetic. The dominant approach was to perform arthrocentesis before administering the appropriate injection substance (I-PRF, PRP, HA, or NSAID). Two current studies on the intra-articular administration of NSAIDs, specifically tenoxicam and piroxicam, are noteworthy. A mixture of PRP and HA was injected in another two trials. These two innovative approaches may prove to be significant directions for further research on injection treatment of TMJs.

## 1. Introduction

### 1.1. Background

Temporomandibular disorders (TMDs) are a group of conditions manifesting themselves, among others, with temporomandibular joint (TMJ) pain and limited mandibular abduction [1]. TMDs affect between 7% and 31% of the population and it is very possible that this percentage will only increase in the coming years [2,3,4,5]. In addition to somatic ailments, TMDs may also result in social exclusion, chronic anxiety and stress, and depression [6]. People suffering from TMDs take sick leave from work and a disability pension two to three times more often [7].

Current methods of treating TMD include pharmacotherapy, physiotherapy of the masticatory muscles, splint therapy, arthrocentesis (AC), and administration of substances to the TMJ cavity in the form of intra-articular injections [8,9,10]. However, none of the available methods meets the criteria of a golden mean, hence it is necessary to adapt treatment to a specific case, and if there is no response, implement a different protocol. Intra-articular injections prove effective in articular pain treatment and associated limitation of jaw mobility [11]. Therefore, they are performed primarily in osteoarthritis and internal derangement.

### 1.2. Rationale

Minimally invasive treatment of temporomandibular joints (TMJs) involves inserting an injection needle or needles intra-articularly [11]. Possible interventions include: (1) lavage of the joint cavity, (2) viscosupplementation of hyaluronic acid (HA) as the main component of synovial fluid, (3) administration of drugs, i.e., corticosteroids (CS), (4) injection of blood-derived or adipose-derived autografts, i.e., platelet-rich plasma (PRP) or injectable platelet-rich fibrin (I-PRF), or (5) deposition of an irritant agent, i.e., hypertonic dextrose (HD) [8,12]. Injection techniques are used for the painful limitation of jaw mobility and its hypermobility, although different substances and places of deposition are used depending on the indications [11].

The dynamic development of injection techniques is manifested by the growing number of published clinical studies, the widening range of substances administered, and the increasingly thorough verification of the validity of sticking to established practices. Within the current injection technique development, the issue of the potential possibility of regenerating articular cartilage using autografts is raised [13]. On the other hand, there is increasing attention on the adverse effects of CS and local anesthetics administered intra-articularly [10]. Moreover, it has been considered to replace routine injections into the upper TMJ compartment with lower-compartment ones [14].

Regularly publishing systematic reviews on minimally invasive TMJ treatments allows for collecting and verifying scientific evidence on the effectiveness of individual treatment methods. These are complemented by scoping reviews, summarizing knowledge about specific emerging techniques [9,11,14]. The only mapping review covered the period until March 2023 and has revealed general trends in recent years [8]. The literature lacks synthetic works presenting more recent findings, which we decided to supplement with this rapid review.

### 1.3. Objective

This rapid review aims to identify and summarize the latest primary research on intra-articular injections into the temporomandibular joints, particularly emphasizing novel solutions.

## 2. Materials and Methods

The content and layout of this paper is based on the Virginia Commonwealth University Rapid Review Protocol [15]. This review was registered in the Open Science Framework Register under the following number: J9GTC.

### 2.1. Eligibility Criteria

Randomized clinical trials were included. Dysfunctions limiting the range of motion of the mandible, articular pain without changes in the range of abduction, as well as habitual dislocation were allowed (Table 1). The condition for inclusion in the study was treatment using intra-articular injections. Less invasive co-interventions were allowed, e.g., psychotherapy, physiotherapy, pharmacotherapy, or splint therapy. Studies that performed more invasive procedures, such as arthroscopy or open TMJ surgery, were excluded. Injection of a placebo or other substance, including AC, was accepted as an eligible control. Various co-interventions in the study and control groups excluded a given report from further processing due to the impossibility of objectively comparing the injection component of complex therapy. The change in quality of life, articular pain intensity, and mandibular range of motion was assessed. A minimum follow-up period of 4 weeks was required. Reports available in full text in English were included, following the aim of the work, and only those published in 2023.

### 2.2. Review Protocol

Based on the above eligibility criteria, the following search query was developed: “temporomandibular AND (injection OR intra-articular OR arthrocentesis OR lavage OR rinsing)”. Sources indexed by the Elsevier Scopus, Cochrane Library, and United States National Library of Medicine PubMed search engines were searched.

The main search was conducted on 18 December 2023, and was supplemented by follow-up searches on 4 January 2024 and 20 June 2024. A search engine filter was used to preselect reports published only in 2023. Two researchers (K.C. and M.C.) conducted the selection process. In the first stage, titles and abstracts were screened, then a full-text eligibility assessment was performed. In discrepancies between independent assessments, doubts were discussed until a consensus was reached. The quality of the trials presented in the source reports was appraised (F.B. and K.L.) in terms of the level of evidence according to The Oxford Levels of Evidence 2 scale. In the next step, data from the content of the articles were collected (F.B. and K.L.) and tabulated. If necessary, mathematical transformations and changes in units were made. Missing data were noted and no attempt was made to provide estimates.

## 3. Results

### 3.1. Selection Process

**Figure 1:**
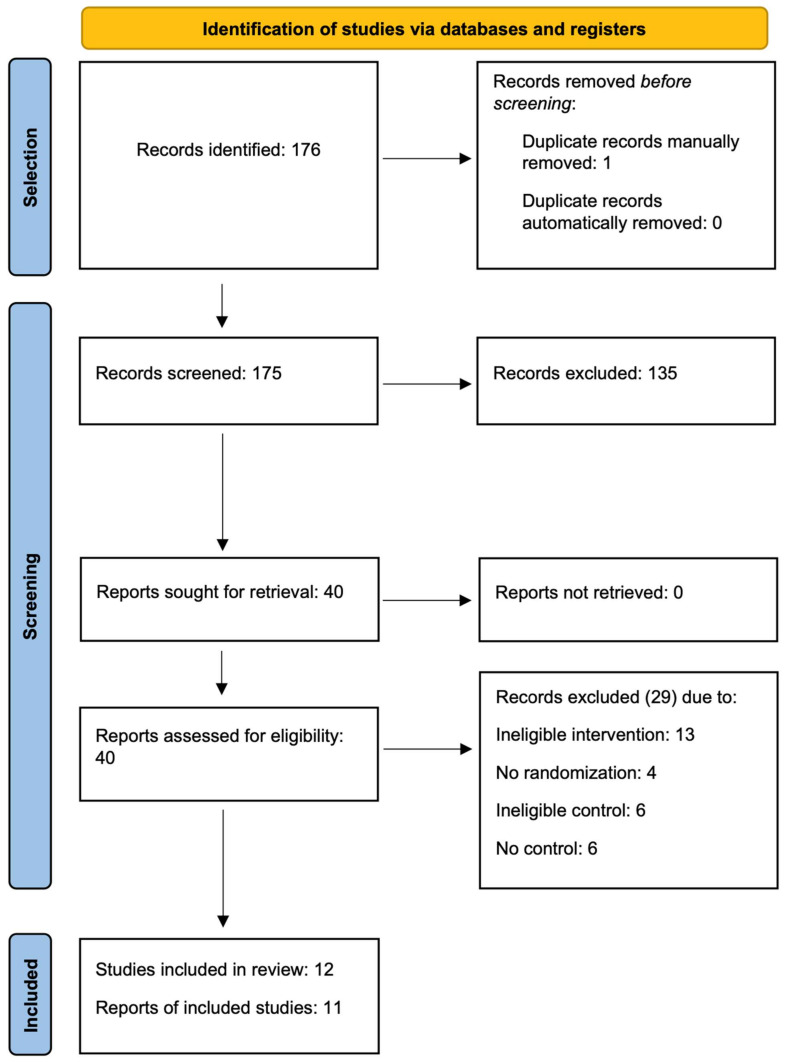
Flow diagram.

The flow diagram in Figure 1 illustrates the subsequent selection stages. Of the 176 records that were identified, 1 of them was removed manually as a duplicate, 135 were excluded at the abstract screening stage. During the full-text evaluation, 29 reports were rejected. The reasons for excluding individual papers are summarized in Table A1. The main reason for rejection was ineligible intervention, including procedures that were more invasive than intra-articular injections, especially arthroscopy. Uncontrolled studies, ones with an inadequate control group, and non-randomized ones were also excluded. Ultimately, 12 reports on randomized controlled trials were included.

### 3.2. Characteristics and Outcomes of Included Studies

Twelve studies on 603 patients in total were included in the synthesis (Table 2 and Table 3). All these studies were of level 2 evidence by The Oxford Levels of Evidence 2 scale. The sample size ranged from 14 to 91 and had a mean value of 50.3, and a median of 60. Diagnoses were highly heterogeneous, from general ones such as TMDs, to specific ones such as disc displacement with or without reduction. The qualified reports also included the treatment of recurrent TMJ dislocation. In this diagnosis, injection into the TMJ was intended to limit jaw mobility, which opposed the effect expected in the other studies. Follow-up time in various studies ranged from a month up to 12 months.

The collected material included research on substances routinely used for articular pain and mandibular hypomobility. These were rinsing solutions, HA, and centrifuged autologous blood products. A hypertonic dextrose (HD) solution was used in recurrent TMJ dislocation.

Several less common treatment protocols were reported, i.e., intra-articular administration of tenoxicam, piroxicam, and a mixture of HA with PRP. The first two substances are non-steroidal anti-inflammatory drugs. Administering drugs inside the TMJ is not a unique idea in itself. CS have been particularly popular and are gradually being abandoned in favor of other approaches [8]. A predominant contribution of CS was observed in the reported adverse events following intra-articular administration to the TMJ [9,10]. However, non-steroidal anti-inflammatory drugs are still poorly researched in the intra-articular route of drug administration.

Both HA and PRP are recognized substances used for TMJ articular pain and limitation of mandibular abduction. Their combination is not routinely used and may prove beneficial. The research on this topic is innovative and therefore fits into the purpose of this review.

## 4. Discussion

Injection treatment is a dynamically developing group of therapeutic methods in TMD management [8]. This is due to its minimal invasiveness and proven quick effectiveness in relieving joint pain and limited jaw mobility [26,27]. The only mapping review in this field showed an increased reporting of studies on autologous preparations [8]. In connection with the results of this rapid review, current interest in non-steroidal anti-inflammatory drug administration is also noticeable [8].

AC as a monotherapy is a recognized method of treating articular pain and hypomobility [26]. It involves injecting the TMJ cavity and depositing the irrigation fluid within [26]. Several types of AC have been described and used, including the double-needle technique, single-needle technique (using a double-lumen needle), and pumping technique [19,26]. The first two involve puncturing the TMJ cavity and gently rinsing it with physiological saline or a ringer lactate flow. The pumping technique consists of repeated fluid administration and removal through the same route. AC is intended to rinse out the inflamed synovial fluid from the joint cavity. The pumping technique additionally exerts pressure on the joint surfaces, which causes the disintegration of inflammatory tissue and facilitates TMJ function [26]. In recent years, it was often proposed to administer various substances to the TMJ after arthrocentesis. These substances were, among others, HA, autologous blood products, or CS. However, sole rinsing of the joint has therapeutic significance. Some clinicians administer injectables into the TMJ without prior AC [11,27]. This matter is still a subject for future scientific research. 

### 4.1. Main Findings

There is a scientific dispute as to whether AC itself is crucial for the treatment of pain and immobility of TMJs, or whether it should be followed by the administration of one of the known injection substances, e.g., HA or a blood derivative. The collected material identified four randomized clinical trials on this topic. The synthesized data is inconsistent and does not prove one specific thesis. Bayramoglu et al. stated that tenoxicam in addition to AC neither relieves pain nor increases mandibular mobility [16]. In turn, Gupta et al. [18] proved that piroxicam combined with AC reduced pain and increased jaw mobility in patients with anterior disc displacement without reduction. Despite the similarity of piroxicam and tenoxicam in terms of structure and pharmacokinetics, the results of the studies are inconsistent. In the context of the lack of previous randomized clinical trials on the administration of piroxicam to the TMJ, further research on this topic is needed [8]. The differences may be due to diagnoses varying between the groups treated in both trials. Another study supporting the claim that adding an injectable increases the effectiveness of AC is the report of Işık et al. [25]. These authors observed that adding I-PRF reduces pain and increases mandibular abduction even further than lavage alone. Sait et al. proved that following AC with HA administration does not influence pain relief, but enhances jaw mobility [12]. Despite the need to perform further randomized studies, it can be noted that a promising direction in the use of additional substances following AC is to consider the use of I-PRF or piroxicam in disk displacement without reduction. Whether the effectiveness of injection AC supplementation results from the diagnosis of disk displacement without reduction, or the use of I-PRF or piroxicam requires clarification in the future. Nevertheless, AC alone is an effective medical procedure that relieves pain and increases mandibular mobility.

Injecting nonsteroidal anti-inflammatory drugs into the TMJ cavities has been gaining popularity in recent years, as reflected in a growing number of clinical trials. However, the validity of such a procedure and the detailed selection of pharmaceuticals are issues that still require stronger scientific support. The included two randomized clinical trials present conflicting results of intra-articular nonsteroidal anti-inflammatory drug injections. Representatives of this drug group, i.e., tenoxicam and piroxicam are similar in structure and pharmacokinetics. Bayramoglu et al. concluded that tenoxicam administration is not beneficial for the clinical effects of AC, neither in terms of changing pain severity nor increasing jaw mobility [16]. Gupta et al. state that piroxicam increases the outcomes of AC in both domains mentioned above [18]. Based on the collected material, it cannot be concluded that combining nonsteroidal anti-inflammatory drugs with AC has a positive or negative effect on its clinical effects. As mentioned earlier, the key here may be the diagnosis, not the specific representative of the group of non-steroidal anti-inflammatory drugs. Further clinical trials and their meta-analytic comparison are needed.

The results of experimental studies suggest the possibility of regeneration of the cartilage covering the TMJ surfaces. The greatest hopes for the actual clinical use of this potential are associated with autologous transplants. This group of substances includes self-derived preparations of adipose tissue, rich in mesenchymal stem cells, and, easier to obtain in an outpatient setting, centrifuged preparations from the patient’s blood. The latter group has many representatives, including primarily plasma rich in growth factors (PRGF), PRP, and I-PRF. The multitude of preparations differing in their composition makes scientific syntheses difficult. Treated separately, they are represented by rather scanty clinical material, albeit collective meta-analyses seem pointless given the significant differences between individual representatives of this group. Clinical comparison of these substances is desirable in the current state of knowledge because it could lead to the displacement of weaker-effect compositions by those with superior therapeutic efficacy. Sharma et al. proved the superiority of I-PRF over PRP in reducing articular pain and increasing maximum mouth opening [23]. These results indicate the advantage of one substance over the other, which, in connection with future similarly designed studies, may contribute to replacing the less effective composition with a better one.

### 4.2. Novel Solutions

In the systematic review, we identified articles describing innovative solutions in temporomandibular disorder treatment. In the paper of Hegab et al., the study group treatment was based on the use of AC + PRP + HA and compared with AC + PRP and AC + HA controls [20]. The administration of PRP in combination with HA after AC is novel and deserves further attention. The study’s results were noteworthy as the use of PRP + HA in combination with AC resulted better in: (1) increasing maximum voluntary mouth opening, (2) reducing pain, and (3) improving joint sounds, compared to AC + HA. This conclusion was based on a 12-month follow-up, considered a long-term observation in injectable TMJ treatment trials.

Attia et al. compared a group treated with a combination of HA and PRP with HA and CS controls [22]. Hence, the cited study has a major limitation i.e., no reference point of a well-studied TMD treatment technique when comparing two innovative approaches. It would be beneficial to conduct subsequent clinical trials comparing HA and PRP with a well-known and widely used injection treatment for TMD. Attia et al. concluded that HA and PRP relieved TMJ pain better than HA and CS over a 6-month perspective [22]. It was noteworthy that in the 1-week perspective, the combination of HA and CS gave better outcomes, which may be due to the strong anti-inflammatory effect of the drug.

### 4.3. Limitations of the Evidence

The selected primary studies vary in specific diagnoses, sample sizes, injectables, and treatment protocols.

### 4.4. Limitations of the Review Process

Due to the rapid nature of this review, it supplemented the state of knowledge with the latest findings. The results of older studies were intentionally not included. Therefore, the syntheses conducted in this review cannot be treated as a contribution to therapeutic guidelines (classical systematic reviews are used for this purpose). Instead, the conducted syntheses indicate the current directions of scientific research and summarize their results. Furthermore, this review was limited to English-language and well-indexed sources.

## 5. Conclusions

This rapid review complemented the only mapping review on injection treatment of temporomandibular joints. Clinical trials on injection treatment of TMJs are still highly heterogeneous in terms of the substances used. Currently, the choice of autologous blood products or HA as the active injection substance is the standard. Innovative research on mixing the above injectables is promising. The hitherto poorly studied intracavitary administration of nonsteroidal anti-inflammatory drugs has been evaluated in subsequent clinical trials. The presence of new randomized clinical trials on the use of this group of substances encourages a systematic review. Most of the intra-articular administrations are currently preceded by AC.

## Figures and Tables

**Table 1:** Eligibility criteria.

	Criteria for Inclusion	Criteria for Exclusion
Patients	Temporomandibular disorders	Cadaver and animal studies
Intervention	Intra-articular injection	Arthroscopy or open surgery
Comparison group	Placebo or injection of other substance	Co-interventions other than in the study group
Outcome of interest	Quality of life, articular pain, mandibular mobility	None
Time	At least 1 month of follow-up	Other interventions during follow-up

**Table 2:** Study characteristics.

First Author	Number of Patients	Diagnosis	Intervention	Control	Follow-Up
Sait [12]	20	TMD	AC + HA	AC	3 months
Bayramoglu [16]	30	TMJ OA	AC + Tenoxicam	AC	6 months
Dhiman [17]	91	TMJ internal derangement	AC + HA	AC + CS	6 months
Gupta [18]	22	DDwoR	AC + Piroxicam	AC	4 months
Bhargava (Single/double puncture) [19]	40	DDwoR	Double-puncture AC	Single-puncture AC	1 month
Hegab [20]	60	TMJ OA	AC + PRP	AC + HA	12 months
Hegab [20]	60	TMJ OA	AC + HA + PRP	AC + HA	12 months
Bhargava (Heavy bupivacaine) [21]	60	TMJ hypermobility	Bupivacaine + HD	AB	12 months
Attia [22]	60	DDwR	HA + PRP	HA + CS	6 months
Sharma [23]	14	TMJ internal derangement	AC + I-PRF	AC + PRP	9 months
Liu [24]	70	TMJ OA	PRP	HA	6 months
Işık [25]	76	DDwoR	AC + I-PRF	AC	12 months

AB—autologous blood (unprocessed); AC—arthrocentesis; CS—corticosteroids; DDwoR—disc displacement without reduction; DDwR—disc displacement with reduction; HA—hyaluronic acid; HD—hypertonic dextrose; I-PRF—injectable platelet-rich fibrin; OA—osteoarthritis; PRP—platelet-rich plasma; TMD—temporomandibular disorder; TMJ—temporomandibular joint.

**Table 3:** Outcomes.

First Author	Quality of Life	Articular Pain	Mandibular Mobility
Sait [12]	AC + HA shows better outcomes than AC in TMD	AC + HA shows no better outcomes than AC in TMD	AC + HA shows better outcomes than AC in TMD
Bayramoglu [16]	No data	AC + Tenoxicam shows no better outcomes than AC in TMJ OA	AC + Tenoxicam shows no better outcomes than AC in TMJ OA
Dhiman [17]	No data	AC + HA shows better outcomes than AC + CS in TMJ internal derangement	AC + HA shows better outcomes than AC + CS in TMJ internal derangement
Gupta [18]	AC + Piroxicam shows better outcomes than AC in Anterior DDwoR	AC + Piroxicam shows better outcomes than AC in Anterior DDwoR	AC + Piroxicam shows better outcomes than AC in Anterior DDwoR
Bhargava (Single/double puncture) [19]	Single-puncture AC has similar outcomes to Double-puncture AC	Single-puncture AC and Double-puncture AC show significant improvement	Single-puncture AC and Double-puncture AC show significant improvement
Hegab [20]	No data	PRP mixed with HA decreases pain more than PRP or HA alone	PRP mixed with HA increases MVMO more than PRP or HA alone
Bhargava (Heavy bupivacaine) [21]	No data	Heavy Bupivacaine-Dextrose Prolotherapy reduces TMJ pain comparably to AB	Heavy Bupivacaine-Dextrose Prolotherapy reduces MIO comparably to AB
Attia [22]	No data	HA mixed with PRP has much better effects in long-term follow ups (6-month perspective) than HA mixed with CS; HA mixed with CS have better outcomes in short-term follow-up (1 week) than HA mixed with PRP	HA mixed with PRP presented similar outcomes to HA mixed with CS
Sharma [23]	No data	AC mixed with I-PRF decreases pain more than AC mixed with PRP	AC mixed with I-PRF increases MMO more than AC mixed with PRP
Liu [24]	PRP increases quality of life similarly to HA	PRP has much better outcomes in short-term (1-month perspective) follow-up than HA	PRP has much better outcomes at 1 month, 3 months, and 6 months than HA
Işık [25]	No data	AC mixed with I-PRF decreases pain more than solely AC over 12 months post-intervention	AC mixed with I-PRF increases MMO more than solely AC over 12 months post-intervention

AB—autologous blood (unprocessed); AC—arthrocentesis; CS—corticosteroids; DDwoR—disc displacement without reduction; HA—hyaluronic acid; I-PRF—injectable platelet-rich fibrin; MIO—maximum incisal opening; MMO—maximum mouth opening; MVMO—maximum voluntary mouth opening; OA—osteoarthritis; PRP—platelet-rich plasma; TMD—temporomandibular disorders; TMJ—temporomandibular joint.

## Appendix A

**Table A1:** Excluded reports.

First Author	Digital Object Identifier	Exclusion Reason
Speculand [28]	10.1016/j.bjoms.2022.10.001	Exclusion reason
Raveggi [29]	10.23736/S2724-6329.22.04653-8	No control
Dinsdale [30]	10.1016/j.msksp.2023.102756	No control
Vingender [31]	10.1016/j.jcms.2023.01.017	Ineligible control
Bilgir [32]	10.1080/08869634.2020.1853889	Ineligible control
Keshavamurthy [33]	10.1097/phm.0000000000002118	Ineligible control
Gaete [34]	10.1007/s10006-023-01158-2	No control
Rajpoot [35]	10.1007/s12663-021-01627-9	Ineligible intervention
Araidy [36]	10.3290/j.qi.b4007423	Ineligible intervention
Ângelo [37]	10.1016/j.ijom.2023.06.008	Ineligible intervention
Rodrigues [38]	10.1016/j.jcms.2023.01.006	Ineligible intervention
Wu [39]	10.3390/jcm12041657	Ineligible intervention
Schabrun [40]	10.1016/j.ynpai.2023.100117	Ineligible intervention
Li [41]	10.1007/s11282-022-00621-2	Ineligible intervention
Ali [42]	10.1097/scs.0000000000009820	Ineligible intervention
Erdil [43]	10.1016/j.jormas.2023.101438	Ineligible intervention
Martins [44]	10.1016/j.jcms.2023.02.001	Ineligible intervention
Ângelo [45]	10.1016/j.jcms.2023.09.010	Ineligible intervention
Navaneetham [46]	10.1007/s12070-023-03890-3	Ineligible intervention
Mohammed [47]	10.26355/eurrev_202306_32605	Ineligible intervention
Symanski [48]	10.1007/s00256-022-04225-z	No control
Navaneetham [49]	10.1007/s12663-023-01986-5	No control
Pohranychna [50]	10.36740/wlek202301121	No randomization
Tang [51]	10.1016/j.ijom.2022.08.018	No control
de Almeida [52]	10.3390/jcm12093228	Ineligible control
Cömert Kiliç [53]	10.1016/j.joms.2022.12.023	Ineligible control
Fayed [54]	10.1016/j.jormas.2023.101644	Ineligible control
Hassan [55]	10.1097/scs.0000000000009085	No randomization
Taşkesen [56]	10.1080/08869634.2020.1861887	No randomization
